# Clinical and pathological characteristics of pulmonary meningioma: a case report and literature review

**DOI:** 10.1177/03000605241293675

**Published:** 2025-02-21

**Authors:** Hui-Fang Chang, Nuerdong Maimaitiaili, Jun-Feng Huo, Zhu-Lei Sun

**Affiliations:** 1 Department of Pathology, The Sixth Hospital of Wuhan, Affiliated Hospital of Jianghan University, Wuhan, China; 2 Department of Neurosurgery, Shanghai Donglei Brain Hospital, Shanghai, China

**Keywords:** Histology, lung, primary pulmonary meningioma, immunohistochemistry, surgery, case report

## Abstract

This case report aimed to examine the clinical pathological characteristics, immunohistochemical phenotype, and differential diagnosis of primary pulmonary meningioma (PM), which is a rare tumor. A retrospective analysis was conducted on the clinical data, imaging manifestations, histological features, immunohistochemical results, and *in situ* hybridization results of a 60-year-old male patient who underwent surgical resection and was diagnosed with PM by pathology. Additionally, the relevant literature was reviewed. Multiple nodules were detected in the right lung of the patient during a re-examination because of a novel coronavirus infection but there were no obvious clinical symptoms. Imaging revealed well-defined masses in the upper, middle, and lower lobes of the right lung, and the masses were surgically removed. Microscopy showed that the boundary between the tumor and surrounding lung tissue was clear, and consisted of spindle cells and epithelioid cells. The final diagnosis was multiple grade II meningiomas of the right lung. No postoperative radiotherapy or chemotherapy was performed. There was no recurrence or metastasis during a 6-month follow-up. Pulmonary metastatic meningioma is rare, while primary PM is even rarer. Surgical resection is the preferred treatment method for PM, with a generally good prognosis, but a few malignant manifestations may require close follow-up.

## Introduction

Pulmonary meningioma (PM) is a rare tumor, which was first reported by Kemnitz et al.^1^ in 1972. PM is the same as a meningeal epithelial (arachnoid) cell tumor, which occurs in the dura mater of the central nervous system, but there is no major central nervous system lesion. The mechanism of PM is still unclear but may be related to a residual neural crest in the embryonic stage, trauma, infection, hormone concentrations, or genetic factors.^2^ PM is mostly benign and usually manifests as solitary nodules. Multiple nodules are extremely rare. PM grows slowly but there are also a few malignant manifestations that are invasive and metastatic.^3^ We report a clinical pathological analysis of a PM case combined with a literature review to improve understanding of this disease. The reporting of this study conforms to the CARE guidelines.^4^

## Case presentation

### Patient’s information

A male patient in his early 60s with coronavirus disease 2019 visited the Pathology Department of Wuhan Sixth Hospital. Clinical data, imaging manifestations, histological features, immunohistochemistry results, and *in situ* hybridization test results were collected and followed up. The patient had no obvious clinical symptoms. A physical examination revealed no abnormalities. A computed tomography scan in our hospital showed multiple nodules in his right lung after having a computed tomography scan in another hospital owing to the coronavirus infection.

### Clinical findings

#### Laboratory and imaging examinations

**Figure 1.**
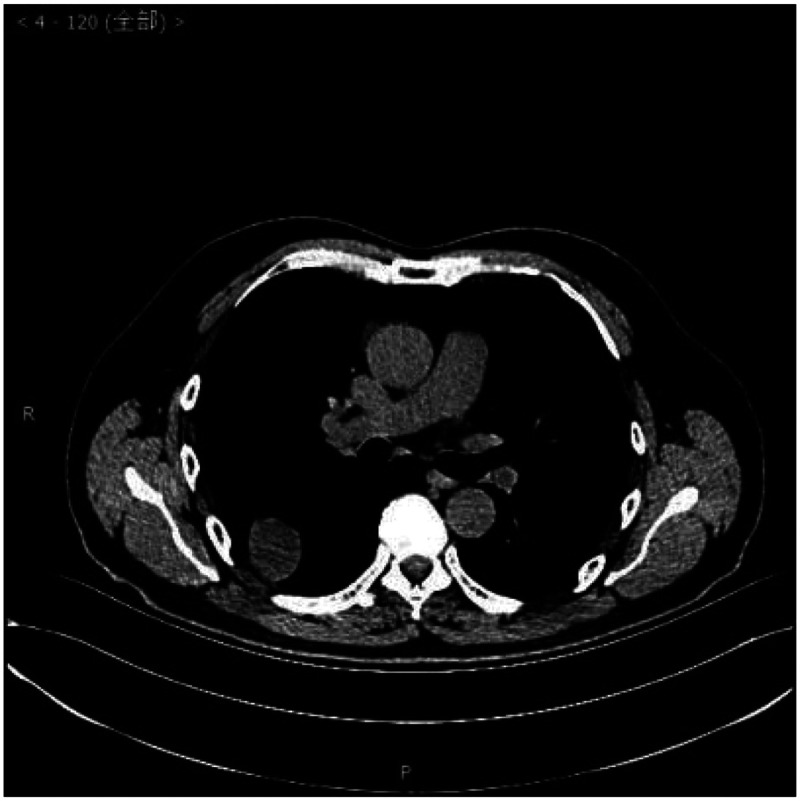
Chest computed tomography scan.

**Figure 2.**
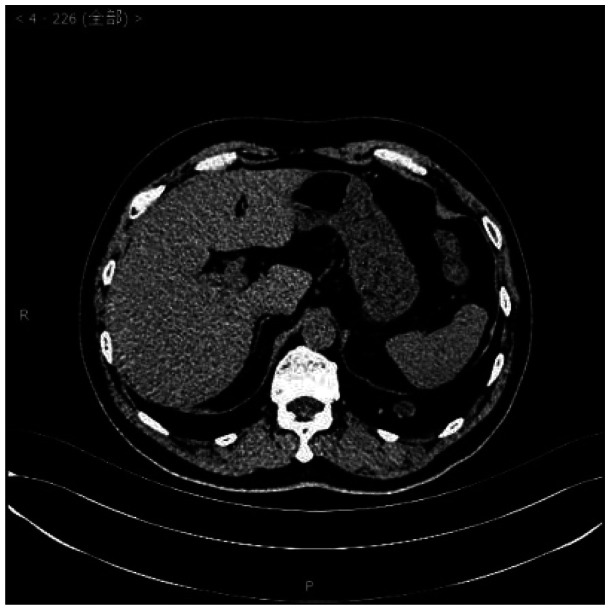
Chest computed tomography scan.

**Figure 3.**
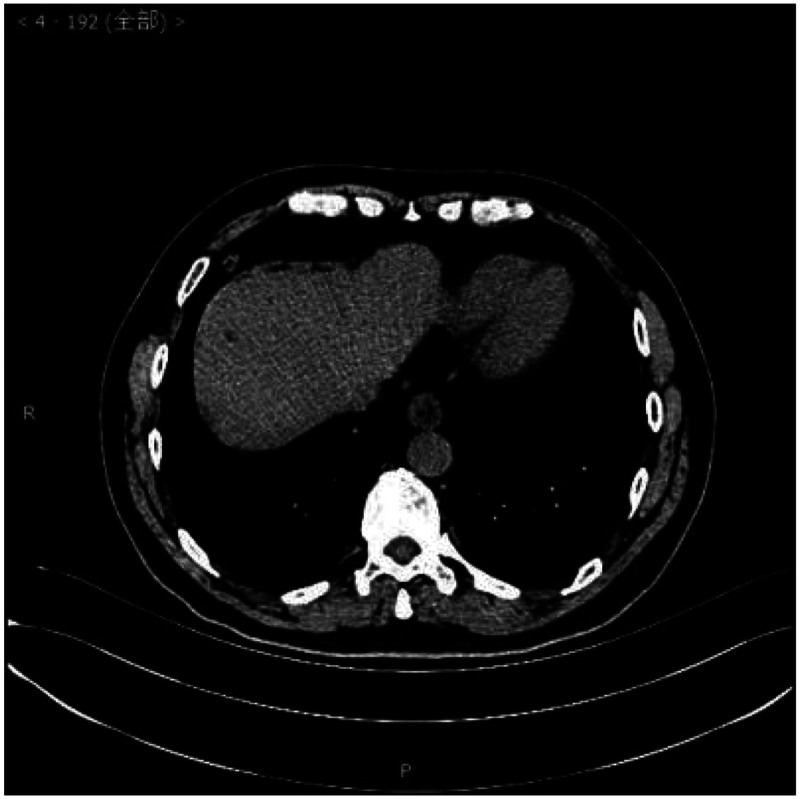
Chest computed tomography scan.

Laboratory examinations such as blood tests, urine tests, liver function, kidney function, and arterial blood gas analysis were within normal limits. A chest computed tomography scan showed a circular nodular shadow in the posterior segment of the right upper lobe measuring approximately 3.5 × 3.2 × 2.5 cm, with clear edges and uniform density. A circular nodular shadow in the middle lobe of the right lung measured approximately 2 × 1.7 × 0.9 cm, with clear edges and uniform density. Another circular nodular shadow in the lower lobe of the right lung measured approximately 0.7 ×0.6 × 0.6 cm, with clear edges and uniform density. No other tumors or enlarged lymph nodes were found (Figures 1–3). No abnormalities were found in cranial magnetic resonance imaging. A bronchoscopic biopsy and immunohistochemistry were not able to provide a clear diagnosis owing to limited tissue samples. Taking into consideration multiple nodules in the right lung, the differential diagnosis included pulmonary metastasis, tuberculosis, pulmonary mycosis, and pulmonary amyloidosis. Right upper, middle, and lower lobe mass resection was performed surgically under thoracoscopy.

#### General observations

During surgery, the following observations were made. In the upper lobe of the right lung, the size of the tissue was 9 × 4 × 3 cm. A 3.5 × 3.2 × 2.5-cm nodule was found in the subcapsular layer 1.5 cm from the incision edge. After the nodule was resected, the lung tissue was sutured using metal wire. The nodule was gray-white with a soft and clear boundary. In the middle lobe of right lung, the size of the tissue was 5.5 × 3.5 × 0.9 cm. A 2 ×1.7 × 0.9-cm nodule was found in the subcapsular layer 1 cm from the incision edge. After the nodule was resected, the lung tissue was sutured using metal wire. The nodule was brown-white with a soft and clear boundary. In the right lower lobe of the lung, the size of the tissue was 6 × 2.5 ×0.8 cm. A 0.7 × 0.6 × 0.6-cm nodule was found in the subcapsular layer 0.5 cm from the suture edge. After the nodule was resected, the lung tissue was sutured using metal wire. The nodule was gray-brown with a soft and clear boundary.

#### Microscopic observations

**Figure 4.**
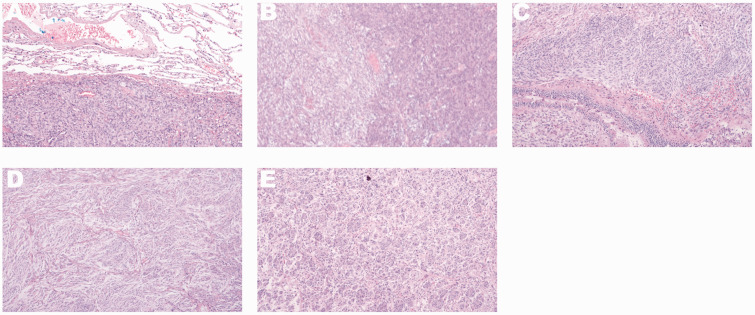
Microscopic observation. (a) The boundary is clear between the tumor and surrounding lung tissue. (b) The tumor is composed of spindle-shaped cells and epithelioid cells. (c) Epithelial cells are distributed in a nested pattern, partially located in the center of spindle cell vortices, and in conjunction with spindle cells, they form a granular structure of the arachnoid membrane. (d) Spindle-shaped cells are arranged in bundles and weaves, with a swirling structure surrounding the central blood vessel and (e) sand-like particles are visible.

Microscopy showed a clear boundary between the tumor and surrounding lung tissue, consisting of spindle cells and epithelioid cells. Epithelial cells were distributed in a nest-like manner, and some of these cells were located in the center of spindle cell vortices, forming a granular structure of the arachnoid membrane in conjunction with spindle cells. Spindle-shaped cells were arranged in bundles and weaves, with a swirling structure surrounding the central blood vessel. Calcification and psammoma bodies were observed (Figure 4(a)–(e)).

#### Immunohistochemistry and in situ hybridization

**Figure 5.**
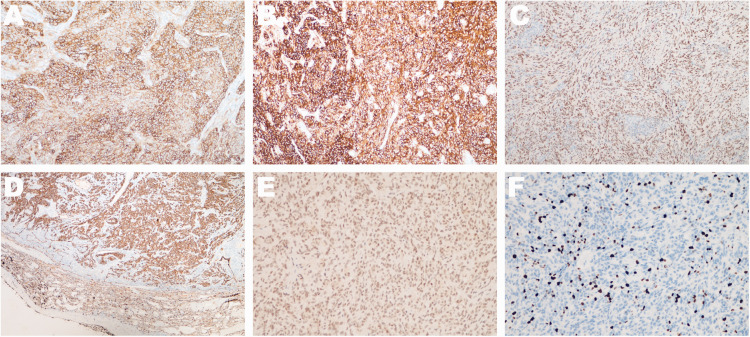
Immunohistochemistry. (a) Somatostatin receptor 2 (short-pass filter, ×200); (b) epithelial membrane antigen (short-pass filter, ×200); (c) Progesterone receptor (short-pass filter, ×200); (d) phosphoenolpyruvate carboxykinase (short-pass filter, ×100); (e) BAP1 (SP, ×200) and (f) Ki67 (SP, ×200).

The epidermal component of the masses showed the following: somatostatin receptor 2 (SSTR2), strong+; progesterone receptor (PR)−; epithelial membrane antigen (EMA), strong+; D2-40, strong+; phosphoenolpyruvate carboxykinase (PCK)+; cytokeratin (CK) 8/18+; and the KI67 index, 5%. The spindle-shaped component of the masses showed the following: SSTR2+; PR+; EMA+; D2-40+; PCK−; CK8/18 part+; and the KI67 index, 1% (Figure 5(a)–(f)). Other indicators, namely CD34, smoot muscle actin (SMA), desmin, S100, anaplastic lymphoma kinase, CD23, CD35, claudin-4, CK7, CK20, villin, CK5/6, thyroid transcription factor-1 (TTF-1), P40, and Wilms’ tumor gene were negative. Calretinin and BAP1 were positive. *In situ* hybridization showed that that Epstein–Barr virus-encoded small RNA was negative. The final diagnosis was multiple grade II meningiomas of the right lung according to the World Health Organization classification.

### Therapeutic intervention and follow-up

No radiotherapy or chemotherapy was performed. No recurrence or metastasis was observed after 6 months of follow-up.

## Discussion

PM is a rare tumor, with an incidence rate of approximately 0.01% to 0.2%.^5^ The mechanism of PM is still unclear and may be related to a residual neural crest in the embryonic stage, trauma, infection, hormone concentrations, or genetic factors.^2^ PM is mostly benign,^6^ with slow growth and a good prognosis, but there are also a few malignant manifestations that are invasive and metastatic.^3^ The clinical manifestations of PM are not typical, and most patients have no symptoms or only mild respiratory symptoms, such as cough, expectoration, and chest tightness. Studies have reported that an imaging examination is the main method of detecting PM.^7^ PM often manifests as isolated or multiple well-defined masses with uniform or uneven density, and may have calcification or bleeding. Multiple PMs in the lungs are extremely rare, with only a few cases reported.^8^ When imaging cannot be qualitative, some doctors do not perform a lung biopsy for a qualitative analysis. Additionally, because of difficulty in localization or small samples, there is a certain risk, and thus using fine-needle aspiration is not easy for a diagnosis.^9^ In our case, the patient underwent lung mass resection after undergoing a bronchoscopic biopsy. Tumors were observed in the upper, middle, and lower lobes of the right lung, with varying sizes. There was no evidence of a primary central nervous system tumor shown by positron emission tomography-computed tomography. After surgical resection and a pathological analysis, the patient was diagnosed with PM.

A histological examination is important for the diagnosis of PM because of the similarity between PM and central nervous system meningioma. The histological characteristics of PM are mainly composed of spindle cells and epithelioid cells. The epithelioid morphology is distributed in a nest-like manner, with a circular or ovoid nucleus and abundant cytoplasm. Spindle cells are arranged in bundles or braids, and the nucleus is slender and elongated. Two forms can coexist or exist alone. Calcification and psammoma bodies can be seen. The immunohistochemical markers of PM mainly include EMA, vimentin, PR, and SSTR2. EMA is one of the most sensitive and specific biomarkers, which can display bridging junctions between tumor cells. Vimentin is one of the specific biomarkers of meningeal epithelial cells. PR and SSTR2 have high positive rates in central nervous system meningiomas, and also have high positive rates in PM.^10,11^ The tumor cells in our patient were positive for EMA, vimentin, PR, and SSTR2, which are consistent with the immunohistochemical characteristics of PM. *In situ* hybridization detection of EBER can exclude lymphoproliferative diseases. Under electron microscopy, the tumor cells of PM show finger-like protrusions and desmosome junctions,^12^ but in this case, electron microscopy was not performed.

PM needs to be differentiated from the following types of tumors. (1) Pulmonary metastatic meningioma is often accompanied by evidence of primary central nervous system tumors, and often multiple tumors occur. The histological characteristics of pulmonary metastatic meningioma are the same as those of primary PM, but immunohistochemical testing shows that the tumor cells of pulmonary metastatic meningioma are positive for lung cancer-related markers such as CK7, creatinine kinase, and TTF-1, and can be distinguished from PM.^13^ There was no evidence of primary central nervous system tumors in our patient, and the tumor cells were negative for lung cancer-related markers, such as CK7, CK20, and TTF-1, which ruled out the possibility of pulmonary metastatic meningioma. (2) Invasive lung adenocarcinoma can manifest as solitary or multiple masses, similar to PM. However, the histological features of lung-infiltrating adenocarcinoma include visible acini, with adherent, papillary, solid, micropapillary, and gelatinous forms. Immunohistochemical testing shows that tumor cells in infiltrating adenocarcinoma of the lungs are positive for lung cancer-related markers, such as CK7, napsin A, and TTF-1, while PM is negative for these markers.^14^ The tumor cells in our patient showed no characteristics of adenocarcinoma cells, and immunohistochemistry also ruled out the possibility of lung-infiltrating adenocarcinoma. (3) Other mesenchymal tumors originating from the lungs, such as leiomyomas, fibromas, and schwannomas, can also manifest as solitary or multiple masses. However, the histological characteristics of these tumors are different from those of PM. Immunohistochemical testing shows that the tumor cells of these mesenchymal tumors are positive for mesenchymal markers, such as SMA, desmin, and S100, while these markers are negative in PM.^15^

The main treatment for PM is surgical resection, which generally does not require radiotherapy or chemotherapy. PM is mostly benign, with a good prognosis after surgical resection and a low recurrence rate. However, there are also a few malignant manifestations, such as atypical or anaplastic meningiomas, which show high atypia, a high proliferation index, and a high number of mitotic figures, as well as invasion of adjacent tissues or distant metastasis.^3^ These malignant PMs need to be closely followed up and treated with radiotherapy, chemotherapy, or targeted therapy according to the situation. Our patient underwent surgical resection of nodules in the upper, middle, and lower lobes of the right lung, and in surrounding normal lung tissue. The pathological diagnosis confirmed PM, World Health Organization grade II. No postoperative radiotherapy or chemotherapy was performed, and there was no recurrence or metastasis during a 6-month follow-up.

In summary, PM is a rare tumor with atypical clinical manifestations. Imaging examinations show isolated or multiple well-defined masses without primary central nervous system lesions, making a clear diagnosis difficult on the basis of imaging and clinical data. Pathological and immunohistochemical examinations are important for the diagnosis of PM. Surgical resection is the preferred treatment method, with a generally good prognosis, but there are also a few malignant manifestations that require close follow-up. There is a small number of cases of PM. Therefore, the mechanism of occurrence of PM still needs to be continuously summarized to provide a reliable theoretical basis for its treatment and prognosis.
